# Conductive Biocomposite Made by Two-Photon Polymerization of Hydrogels Based on BSA and Carbon Nanotubes with Eosin-Y

**DOI:** 10.3390/gels10110711

**Published:** 2024-11-03

**Authors:** Mikhail S. Savelyev, Artem V. Kuksin, Denis T. Murashko, Ekaterina P. Otsupko, Ulyana E. Kurilova, Sergey V. Selishchev, Alexander Yu. Gerasimenko

**Affiliations:** 1 Institute of Biomedical Systems, National Research University of Electronic Technology, 124498 Zelenograd, Russia; nix007@mail.ru (A.V.K.); skorden@outlook.com (D.T.M.); ekaterinaotsupko@mail.ru (E.P.O.); kurilova_10@mail.ru (U.E.K.); selishchev@bms.zone (S.V.S.); gerasimenko@bms.zone (A.Y.G.); 2 Institute for Bionic Technologies and Engineering, I. M. Sechenov First Moscow State Medical University, 119991 Moscow, Russia; 3 World-Class Research Center “Digital Biodesign and Personalized Healthcare”, I. M. Sechenov First Moscow State Medical University, 119991 Moscow, Russia

**Keywords:** two-photon polymerization, conductive composites, biopolymer composites, carbon nanotubes, gelatin, eosin-Y, tissue engineering

## Abstract

Currently, tissue engineering technologies are promising for the restoration of damaged organs and tissues. For regeneration of electrically conductive tissues or neural interfaces, it is necessary to provide electrical conductivity for the transmission of electrophysiological signals. The developed biocomposite structures presented in this article possess such properties. Their composition includes bovine serum albumin (BSA), gelatin, eosin-Y and single-walled carbon nanotubes (SWCNTs). For the first time, a biocomposite structure was formed from the proposed hydrogel using a nanosecond laser, and a two-photon absorption cross section value of 580 GM was achieved. Increased viscosity over 3 mPa∙s and self-focusing with a nonlinear refractive index of 42 × 10^−12^ cm^2^/W make it possible to create a biocomposite structure over the entire specified area. The obtained electrical conductivity value was 19 mS∙cm^−1^, due to the formation of effective electrically conductive networks. For a biocomposite with a concentration of gelatin 3 wt. %, formed by low-energy near-IR pulses, the survival of Neuro 2A nerve tissue cells was confirmed. The obtained results are important for the creation of new tissue engineering structures and neural interfaces from a biopolymer hydrogel based on the organic dye eosin-Y and carbon nanotubes by two-photon polymerization.

## 1. Introduction

Tissue engineering is designed to restore the original functionality of tissue by replacing damaged areas with new tissue during the process of regeneration, which is accompanied by biodegradation of tissue-engineered constructs [1,2]. This technology employs a support framework for volumetric tissue formation, which establishes the requisite conditions for cell proliferation. Neural engineering requires novel materials that can provide specific properties, such as biodegradation [3,4] and electrical conductivity [5,6,7], to enable the transmission of electrophysiological signals during tissue regeneration at the replacement site. Moreover, the materials must possess volumetric properties [8,9,10,11] to ensure comprehensive coverage of the injured area when using scaffold-based three-dimensional cell culture technology. For creation of artificial organs, it is also necessary to have such materials, as they consist of a multitude of tissue types simultaneously [12,13], including those capable of conducting impulses from their point of origin to the contractile region. Composites are the optimal solution for meeting these requirements [7,12,14,15]. The inclusion of specific components in their composition allows for the imparting of particular properties to the composite material. Such composite materials can also be used as neural interfaces for stimulating nerve tissue or to record electrical signals. Neural interfaces should overcome differences between harsh electrical generators and soft tissues and transmit electrical signals with high efficiency [16,17].

Injectable hydrogels are being developed to restore nerve conductivity [18,19,20]. They can be injected directly into target areas to restore conductivity of nerve fibers in the central nervous system, which is difficult due to the inability of neurons to recover from damage. However, in the case of large nerves injuries, when the damage covers a fairly large length, the restoration of their functions is severely limited [21]. For this reason, it is important to develop composite hydrogels capable of supporting the directed growth of nerve cells to ensure the regeneration process.

In neural tissue engineering, hydrogels based on conductive synthetic polymers are actively used, for example, polyaniline (PANi), polypyrrole (PPy), and poly(3,4-ethylenedioxythiophene) (PEDOT). These materials combine high electrical conductivity, flexibility comparable to native biological tissue, and relative biocompatibility. Another advantage is the ability to modify the surface and control properties through chemical modification. Synthetic polymers can act both as the main component of the hydrogel and as a filler in the biopolymer matrix. Table 1 presents the electrical properties and biocompatibility (based on MTT test results) of some materials used in neural tissue engineering to create conductive cell scaffolds.

Despite the advantages of synthetic conductive polymers, they have a number of features that significantly complicate their use in practice. Hydrophobicity and poor solubility negatively affect the cytocompatibility of such a framework. Also, the lack of biodegradation capacity increases the possibility of an inflammatory process at the implantation site and makes it necessary to perform repeated surgical intervention to remove the previously inserted framework [31]. Hydrogels composed of biopolymers warrant considerable attention due to their superior biocompatibility, flexibility, low toxicity, and biodegradability in comparison to hydrogels produced from synthetic polymers [32,33]. However, using only biopolymers, it is not possible to provide the electrical conductivity necessary to stimulate the growth and regeneration of neurons, and it is important to achieve values of the order of 10^−3^–10^−4^ S/cm [29,34]. Therefore, to ensure electrical conductivity, it is promising to use composite hydrogels with a matrix of biopolymers, a conductive filler of metal nanoparticles, and allotropic forms of nanocarbon or nanoparticles of conductive polymers [15,24,26,28,35,36].

To solve neural engineering problems, biocompatible conductive structures can be prepared using different methods. Lyophilization is often used, which allows the creation of bulk materials based on chitosan and multi-walled carbon nanotubes [37], which requires expensive equipment. Another method is evaporation [38]. This method is inexpensive, but its application is limited to the creation of films, for example, based on collagen and carbon nanotubes.

A promising approach to fabricating volumetric composites of the desired shape and size is the method of two-photon polymerization, which can be scaled from micro to macro scale [39,40]. Through the application of short, highly focused pulses, the laser beam achieves an intensity that exceeds the threshold necessary to initiate nonlinear absorption processes [14,41,42,43,44]. These processes result in the formation of cross-links at the site of laser exposure. A variety of hydrogel frameworks based on natural polymers, (collagen [2,41,45], chitosan [1,13], hyaluronic acid [5], bovine serum albumin (BSA) [46,47], etc.) can be polymerized using two-photon polymerization. The partial denaturation or aggregation of BSA molecules observed under the influence of ultraviolet and visible wavelength laser radiation allows the protein to retain its biocompatibility properties [48]. This is due to the presence of reactive groups, including carboxyl, hydroxyl, and sulfonic acid groups, on its surface, which induce differentiation and promote cell adhesion [49,50]. Concurrently, excessive laser exposure at these wavelengths at high power can cause protein denaturation [42,51]. For this reason, the use of low-energy initiating laser radiation of the IR range (1070 nm in the present work) in the two-photon polymerization method is advantageous. Nevertheless, this requires the use of photoinitiators [43,44,52], especially when using lasers with a duration longer than femtoseconds. The eosin-Y photoinitiator has suitable optical characteristics for the specified wavelength range, and it is characterized by the presence of biocompatibility [14,53]. To improve the binding efficiency, functionalizing reagents can be used that are capable of increasing the amount of reacted primary amines (e.g., methacrylic anhydride [54] and furfurylamine [55]) relative to the primary amines present in gelatin.

Most proteins, including BSA, do not have sufficient mechanical strength. To obtain a biocomposite based on BSA, additional components must be used. In this article, gelatin is used to produce a hydrogel [56,57] and single-walled carbon nanotubes (SWCNTs) as a reinforcing framework [15], which makes it possible to obtain samples of arbitrary shape. Such hybrid structures combine the desired properties of proteins with the characteristics of other components.

According to the biocompatibility of BSA, there are a large number of studies that indicate the manifestation of the necessary properties precisely in the case of using a combination of SWCNTs and BSA. The use of such a combination makes it possible to provide sufficient properties for cytocompatibility [58] and hemocompatibility [59] while maintaining strength characteristics. Based on the results of the assessment of the biocompatibility of the SWCNTs and BSA dispersion when mixed with a suspension of mesenchymal stem cells in a culture medium, it was established that with a limited content of carbon nanotubes, there is no significant slowdown in cell growth and their development is not disrupted [60]. The nanotubes are welded together under laser exposure [15,61], which enhances the mechanical strength while maintaining its flexibility. The framework of SWCNTs, appearing during laser exposure, also ensures the electrical conductivity of samples, which is important for signal transmission in neural tissue engineering and in neural interfaces for bioelectronics [62]. As a result of laser photocuring, the strength of the corresponding biocomposites and their degradation time increase, which makes it possible to obtain the necessary characteristics when restoring biotissues [63]. In the case of the formation of biocomposites by the thermostatting method, there is no complete cross-linking of the components, which leads to too rapid degradation of materials based on albumin and nanotubes.

Natural electrical activity corresponding to the electrophysiological characteristics of neurons and connections is ensured by the presence of neural circuits [64]. Electrophysiological and synaptic characteristics depend on the interaction of neurons with the surface, especially when using conductive platforms. The highly conductive substrate inhibits proliferation, while the low conductive substrate has no effect on neuronal behavior. It is the semiconductor platforms that play a useful role as a nerve conductor in achieving neural regeneration, which is achieved through a balance between conductivity and electrical stimulation. The electrical conductivity of cells is provided by the flow of ions circulating through the membrane. Outside the cell, the potential is considered positive, whereas the inside is negative—about −70 mV. This resting potential is maintained in the non-excited state of the cell. However, when an electrical signal occurs, a gradient redistribution of ions occurs, which causes depolarization of the cell. To restore equilibrium (repolarization), the cell increases membrane permeability, ensuring active migration through ion channels.

Electrical stimulation causes polarization of signaling molecules and redistribution of intraplasmic substances, which activates certain cellular processes. The accumulation of calcium ions in the cytoplasm, for example, leads to changes in the concentrations of ATP, glutamic acid, acetylcholine, dopamine, serotonin, and GABA. Studies on neuronal tissue show that electrical stimulation influences the PI3K/AKT/mTOR signaling pathway, thereby preventing cell apoptosis [65]. In addition, the potential difference leads to asymmetry of intracellular signaling pathways and reorganization of microfilaments, which leads to directed cell migration in an electric field—electrotaxis [64].

Electrical stimulation significantly promotes cell differentiation. The effect of the electric current reorganizes membrane receptors and, thus, affects the adsorption of proteins on the membrane surface, causing changes in cell morphology. In combination with changes in signaling pathways, this leads to stimulation of neural differentiation. It is worth noting that differentiation is significantly influenced by the type of stem cells used and the parameters of electrical stimulation [66].

Thus, electrical stimulation is a promising method for improving tissue regeneration via functional restoration of the tissue through differentiation of stem cells, stimulation of their growth and proliferation, inhibition of apoptosis, and fibrosis processes [67,68].

Therefore, in this work we propose the formation of a conductive biocomposite from a hydrogel based on albumin and eosin-Y with a conductive network of carbon nanotubes using the two-photon polymerization method. The use of such biocomposites can be effective for electrical stimulation and, accordingly, for maintaining conditions for the regeneration of nerve tissue.

## 2. Results

### 2.1. Determination of Nonlinear Optical Characteristics

**Figure 1:**
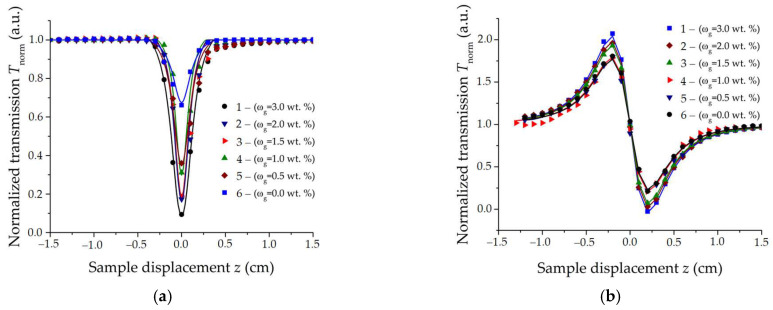
Results of measurements by Z-scan method: (**a**) With open aperture; (**b**) With closed aperture.

The hydrogel is composed of BSA of 20 wt. %, eosin-Y 0.05 wt. %, SWCNTs 0.02 wt. %, and the amount of gelatin for each sample is as follows: 1—3 wt. %, 2—2 wt. %, 3—1.5 wt. %, 4—1 wt. %, 5—0.5 wt. %, 6—0 wt. %. The two-photon absorption cross section was estimated according to the data obtained by the Z-scanning method with an open aperture (Figure 1a) and the nonlinear refractive index in accordance with the results of measurements with a closed aperture. Figure 1b shows graphs of purely refractive Z-scanning. The measurements were carried out under the same conditions. The total energy of a single pulse with duration of 100 ns was 10 µJ at a pulse repetition rate of 50 kHz at a wavelength of 1070 nm. The Z-scan method is a convenient means of choosing a material with optimal characteristics. An important factor influencing the polymerization process is the initial viscosity of the hydrogel, which was measured at a temperature of 24 °C. These results are presented in Table 2.

**Figure 2:**
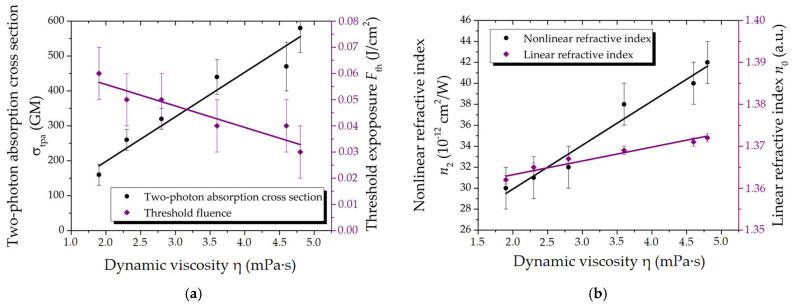
Dependences of the main optical parameters on dynamic viscosity: (**a**) Two-photon absorption cross section and threshold fluence; (**b**) Nonlinear and linear refractive indices.

The value of the two-photon absorption cross section increases with increasing viscosity (Figure 2a), while the value of the threshold exposure of laser radiation decreases. The effect emerges at lower energy parameter values. This characteristic is attributable to the reduction in Brownian motion intensity due to an increase in viscosity (this effect is independent on chemical nature of the particles). Reverse saturable absorption (RSA) occurs under the frequency action of laser radiation. In this case, the effect of saturated absorption (SA) is not observed, for which the absorption of light decreases with increasing light intensity. The results of the studies indicated a tendency for a decrease in the nonlinear refractive index (Figure 2b) at low values of the linear refractive index. At the same time, the viscosity also exhibits a reduction in value. In general, such a change and the positive sign of refractive index indicate a self-focusing effect within the sample. The value of the linear absorption coefficient at a laser wavelength of 1070 nm coincides within the error limits, with the exception of sample 6 (Table 2). Gelatin is absent in this sample, and it is characterized by the formation of a certain amount of sediment from the SWCNTs, which resulted in the lowest value of α = 1.6 cm^−1^.

### 2.2. Determination of Electrical Conductivity of Samples

**Figure 3:**
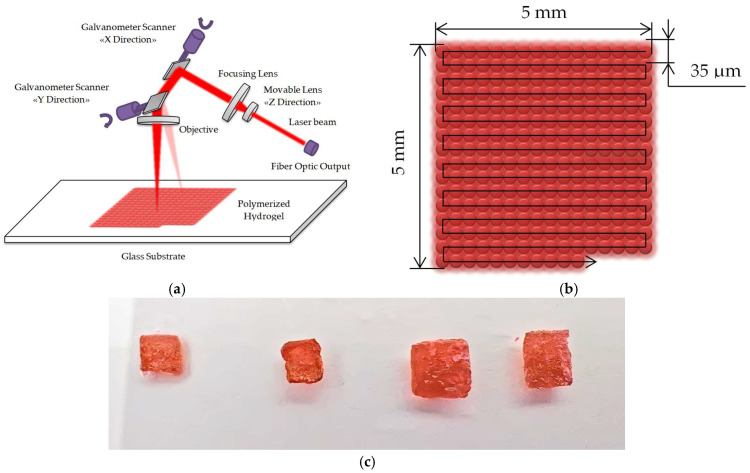
Manufacturing of a biocomposite by two-photon polymerization: (**a**) Laser exposure of hydrogel on a substrate; (**b**) Trajectory of the spot’s movement; (**c**) Appearance of the formed biocomposite (sample 1, 5 layers).

The electrical conductivity was evaluated by the van der Pauw four-probe method. For this purpose, 5 × 5 mm^2^ samples were created based on hydrogel, which is optimal for measurements. Two-photon polymerization was initiated by laser pulses of 100 ns duration with a repetition frequency of 30 kHz at a wavelength of 1070 nm using a fiber laser equipped with a scanning system (Figure 3a). The formed trajectory was a 5 × 5 mm^2^ square. It was taken into account that two-photon polymerization exhibits a localized effect, manifesting only within the zone of sufficient intensity and not across the entire exposure area. The diameter of the focused laser spot was 35 μm. The trajectory shown in Figure 3b was used for uniform exposure over the entire area. The typical appearance of the test samples from hydrogel 1 is shown in Figure 3c. The exposure mode was selected based on the data obtained through Z-scanning, above the threshold exposure of laser radiation for observing two-photon absorption. Since the polymerization process occurs without temperature changes during laser exposure [69], it is suggested that to use the energy region in which the contribution of nonlinear beam attenuation prevails is insufficient for this purpose. By varying the laser exposure above the threshold, the value of the two-photon polymerization threshold is determined. To achieve the corresponding value, it was necessary to use a movement speed of 240 mm/s at a single pulse energy of ~140 µJ. The number of layers varied from two to five with a step of 17 µm. Samples 4–6 exhibited incomplete polymerization. With an adequate value of dynamic viscosity coefficient, the reproducibility is significantly increased, which is confirmed by the production of suitable samples 1–3 for measuring electrical conductivity.

**Figure 4:**
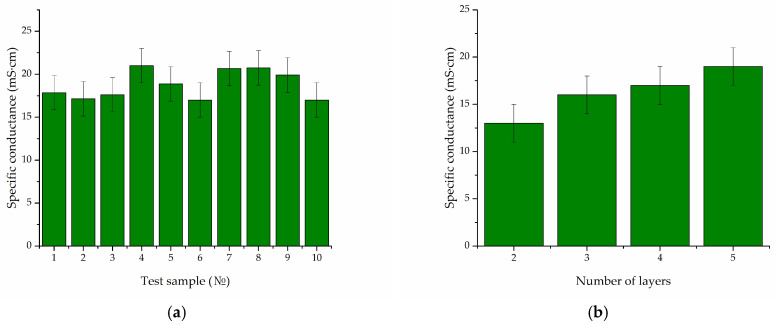
Results of studies of specific resistance of biocomposite 1: (**a**) The value is for 10 printed test samples; (**b**) Dependence of the characteristic on the layer numbers.

Following fabrication, the samples were placed on a dielectric substrate to measure electrical conductivity. Table 3 shows the results of measurements of resistivity and conductivity measurements for samples 1–3. In the case of hydrogel 1, samples with fewer layers ranging from two to four were fabricated. In each case, ten measurements of polymerized biocomposites were performed. Figure 4a shows the results for specific conductance for 10 printed test samples with a constant number of 5 layers for hydrogel 1, while Figure 4b presents the investigation of this value for hydrogel 1 for different layer numbers. Table 3 shows the average values.

The successful fabrication of the biocomposite on the specified 5 × 5 mm^2^ substrate was achieved with the sample with a dynamic viscosity coefficient above 4.6 mPa·s. At lower values, non-polymerized areas are observed. According to the results shown in Table 2 for hydrogels 1–3, the values of two-photon absorption cross section exceed 400 GM, indicating enhanced radiation absorption, which also affects the reproducibility of the result. The lower values of specific conductivity and the highest specific resistivity for biocomposite 3 indicate low SWCNT framework formation. High concentrations of nanotubes above 0.02 wt. % did not lead to a significant increase in electrical conductivity due to the high value of Van der Waals forces, which prevented uniform homogenization of their large quantity in the hydrogel.

### 2.3. Biocompatibility of the Biocomposites

**Figure 5:**
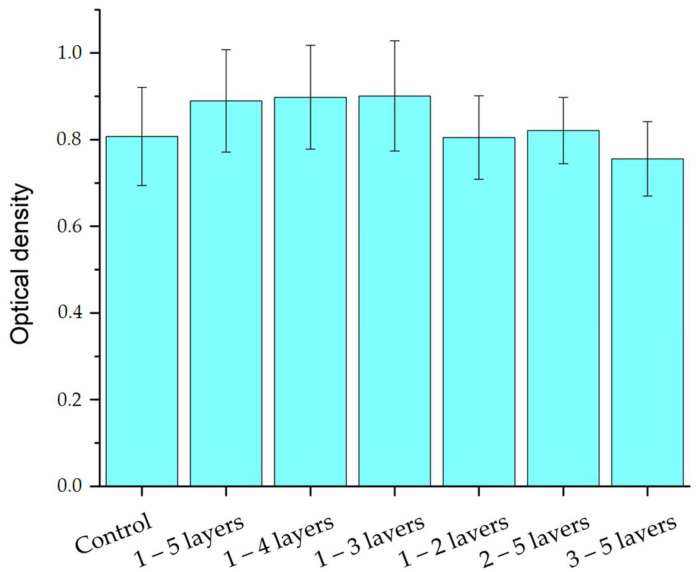
Results of the MTT test of polymerized samples after 72 h of cultivation.

**Figure 6:**
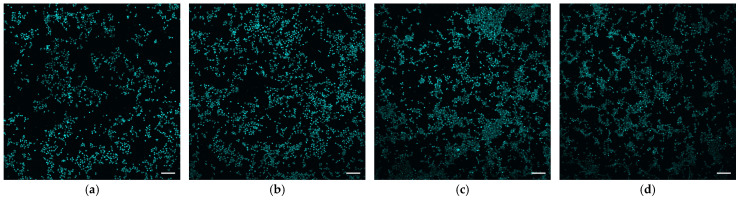
Fluorescence microscopy images of cells after 72 h of cultivation on biocomposite samples: (**a**) Control (clean cover glass); (**b**) Sample 1 (5 layers); (**c**) Sample 1 (4 layers); (**d**) Sample 2 (5 layers). Bar is 100 µm.

Investigations of cell growth during cultivation within 72 h with samples of biocomposite 1 were carried out. The results of the MTT test are shown in Figure 5. With the same number of 5 layers, a slight decrease in cell survival is observed from sample 1 to 3, but such a change in value does not exceed the error limits. This indicates that there is no effect on biocompatibility by changing the amount of SWCNTs relative to gelatin. This is due to the presence of BSA in the biocomposite in addition to gelatin, which together act as a matrix covering the surfaces of SWCNTs. The morphology of the cells was evaluated after the end of their cultivation by staining and viewing on a fluorescent microscope. The resulting microscopic images are shown in Figure 6.

The results of the cellular investigations indicate a positive effect of the samples on cell proliferation. The best cell survival was observed in the samples obtained from three or more layers of sample 1. These samples also have the highest specific conductance. For all the studied biocomposite samples, except for the last sample of the 5 layers of sample **3**, the number of cells exceeds the values in the control. However, for this sample, the optical density values relative to the control are within the permissible values. In vitro studies confirm the growth of cells, which after 3 days cover a significant portion of the samples to form a monolayer. The specified time of the experiment was chosen to prevent the cessation of cell growth and division associated with the lack of free space for growth; in addition, there is a gradual evaporation of the cellular environment over time, which can distort the results [70,71]. This duration of observations over 3 days is often chosen to obtain a reliable result [72,73,74]. At the same time, according to microscopy data, the morphology of the cells in the samples does not differ from the morphology of the cells in the control, which indicates the absence of a toxic effect of the samples. The sample of 5 layers of biocomposite 1 shows the most extensive cell growth and a more uniform distribution of cells over the surface, which can be used to create electrically conductive tissue-engineered structure where complete coverage of the surface with a cell layer is necessary or to create neural interfaces for the repair of nerve tissue.

## 3. Discussion

The creation of an electrically conductive biocomposite for tissue engineering and neural interfaces was based on the use of BSA and SWCNTs. This combination makes it possible to ensure the properties of cytocompatibility and hemocompatibility [15,59]. During laser exposure, a series of processes occur during the formation of the biocomposite in a liquid dispersed medium. One of which is associated with the welding of SWCNTs among themselves, which ensures the formation of stable percolation chains of nanotubes [15,58,61].

Another process is laser-induced crosslinking of proteins [75], which involves partial denaturation (potentially reversible) and aggregation. Aromatic amino acid residues are less susceptible to damage, and no disruption of the polypeptide chain due to fragmentation of protein molecules is observed. Furthermore, to achieve such an effect, it is necessary to use one-photon exposure to ultraviolet (UV) radiation or two-photon exposure in the visible wavelength range, which in some cases allows the formation of materials with BSA without photoinitiators [48,76].

The activation of protein polymerization is facilitated by the use of a photoinitiator that promotes photocrosslinking at relatively low laser power values, as evidenced in references [33,41,42,77]. In this article, eosin-Y was used, which has the required water solubility. It belongs to the free-radical type II photoinitiators, and its use requires a substantial number of amino acid groups on BSA [43]. The cross-linking of dye molecules with proteins occurs under the action of two photooxidation mechanisms. First, the dye molecule is excited, which facilitates the extraction of hydrogen from the BSA molecule (thereby promoting the cross-linking of proteins containing amino acid residues of ketones, phenols, amines, or hydroquinones). The second mechanism is associated with the formation of singlet oxygen, which is capable of reacting with oxidizable amino acid residues to form an electron-deficient protein that unites with an amino acid residue of another protein.

It is also important to understand the processes of interaction between the matrix and filler in biocomposites. Vibrational spectroscopy (FTIR and Raman spectroscopy) allowed us to determine that conformational changes in BSA molecules can occur during laser exposure when albumin interacts with SWCNT. The main mechanisms are reduced to hydrophobic interactions (hydrogen bonds, van der Waals forces, electrostatic forces) and π-stacking. Since the amino acid residues of glutamic and aspartic amino acids located on the periphery of the albumin molecule are polar and have a negative charge at physiological pH values, the radicals of acidic amino acids contain an additional carboxyl group. This group can form chemical bonds not only with the side chains of other amino acids, forming the tertiary structure of proteins. The carboxyl group can chemically bond with carbon nanotubes. Moreover, an increase in the concentration of nanotubes leads to saturation of the nanotube’s functionalization with oxygen atoms of amino acid residues [78]. This results in the emergence of hydrophobic interaction. Raman spectra allowed us to demonstrate the appearance of a significant number of defects in nanotubes in the biocomposite. The defects are caused by the covalent attachment of oxygen to the graphene surface of the nanotubes. An increase in the concentration of nanotubes leads to the saturation of the functionalization of SWCNTs with oxygen atoms of the amino acid residues of glutamic and aspartic amino acids. The mentioned spectral methods confirm the change in amide bands caused by electrostatic attraction between the cationic and carboxyl groups of SWCNTs, which is not observed in the case of anionic biopolymers; some electrostatic repulsion occurs [79]. As a result of light absorption within the eosin-Y molecule, the formation of the eosin radical anion and amine radical cation capable of binding is initiated [53]. Concurrently, SWCNTs are present within the material and these processes affect their welding in the defective areas.

The final component of the aqueous hydrogel is gelatin, the quantity of which was varied in this article. The refractive index is dependent on the amount of gelatin. The positive value of its nonlinear component indicates the occurrence of the self-focusing effect, while this effect increases with increasing dynamic viscosity coefficient. Simultaneously, stable polymerization was not observed at a low concentration of gelatin, which is associated with self-assembly under the action of a laser for a period of approximately 1 ms [42]. In contrast to the almost instantaneous process of radiation absorption, the polymerization process itself requires a certain amount of time. Furthermore, such energy almost does not transfer to the heating of the region, as was shown in experiments with continuous laser radiation [69]. However, sufficient energy is necessary to maintain the polymerization process. With an increase in dynamic viscosity coefficient, the effect on the specified area is more accurate. The exposure by ultrashort pulses with a repetition frequency or pulses of longer duration can be considered precisely as the transfer of the required amount of energy. In this case, the process of two-photon absorption itself is used precisely to transfer the required amount of energy to the specified processes that occur during self-assembly. To induce this process, it is precisely the absorption of radiation that is required, and not its scattering, which is typical for dispersed media with SWCNTs [79], which confirms the significant contribution of the absorption effect in the process of attenuation of the transmitted laser radiation power.

The advantage of two-photon polymerization is the possibility of using low-energy laser radiation in the IR wavelength range. In most cases, expensive ultrashort femtosecond lasers are used to achieve the required energy parameters, but short-duration lasers can also be used [44]. In this article, a nanosecond laser was used for this purpose. At such energy parameters of laser radiation, the use of photoinitiators is necessary, which is used as eosin-Y. In this paper, one of the simplest sets of components is proposed, in particular, gelatin functionalizing reagents (for example, methacrylic anhydride [54] and furfurylamine [55]) can be used to fine-tune the two-photon polymerization process, which can increase the efficiency of amide binding.

## 4. Conclusions

The developed hydrogel composition based on BSA 20 wt. %, eosin-Y 0.05 wt. %, SWCNTs 0.02 wt. %, and gelatin at over 1.5 wt. % exhibits a two-photon absorption cross section of at least 400 GM. When stable polymerization was obtained for a dynamic viscosity of about 3 mPa∙s, sufficient proliferation without morphological disturbance was recorded for all samples. The use of bovine serum albumin promotes the functionalization of the SWCNTs surfaces, and laser-induced cross-linking slows down the degradation. The hydrogel forms a biocomposite under the action of nanosecond laser radiation with a wavelength of 1070 nm. This material is a suitable candidate for use in neural engineering. Electrical conductivity values of at least 17 mSm·cm^−1^ were obtained for this biocomposite. This value is suitable for creating nerve guides that can improve cellular activity with or without electrical stimulation. The combination of its components was selected taking into account biocompatibility, which was proven by results of in vitro studies. The results of the cellular experiments indicate a positive effect of the samples on cell proliferation. The best cell survival from obtained samples was observed in the samples obtained from three or more layers of sample 1. Such biocomposites are also characterized by better electrical conductivity, which makes them suitable for the use of electrical stimulation and, accordingly, for maintaining conditions for regeneration. Thus, the developed samples can successfully be used as structures for neural tissue engineering and neural interfaces for bioelectronics.

Two-photon polymerization technology can be used to directly prepare a biocomposite macrostructure for use as a tissue-engineering structure that has a specific extracellular matrix structure. This is achieved due to the scalability of two-photon polymerization for large-scale applications. The stability of the structure and the degree of organic crosslinking correlate with the dynamic viscosity. Even in the case of using low-energy IR, it is important to use the lowest possible power value, which avoids the formation of burnt areas; in addition, excessive energy of the incident radiation can also lead to particles leaving the hydrogel at the site of action at low dynamic viscosity. The work demonstrates the possibility of scaling two-photon polymerization for large-scale applications, which confirms the possibility of using this technology to create macrostructures for clinical use in neuronal engineering. In this cost-effective method, the proposed material can be used to form nerve guides that can improve cellular activity with or without electrical stimulation. When using stimulation, the best effect of nerve regeneration is achieved when it is applied in the direction of nerve alignment. These developments are important for the creation of a new generation of commercial material for the formation of nerve conduction channels that can accelerate and improve nerve regeneration. Providing conditions for nerve regeneration is important for improving the quality of life of people suffering from neural conduction disorders.

## 5. Materials and Methods

### 5.1. Preparations and Characterization

The preparation of a hydrogel for two-photon polymerization requires the use of a set of components and, thus, the manufacturing process is divided into separate stages. Initially, 100 mL of distilled water is used, to which BSA (BioClot, Aidenbach, Germany) is added under constant mixing with a magnetic stirrer to achieve a concentration of 30 wt. %. A similar procedure was used to prepare gelatin (PanReac Applichem, Barcelona, Spain) to achieve 15 wt. %.

SWCNTs «OSUNT90A^®^» (Carbon CHG, Chernogolovka, Russia) with a diameter from 1.4 to 1.6 nm in the length range from 0.5 to 1.5 μm [80] were prepared separately in a flask with V = 40 mL of distilled water using ultrasonic treatment with a Soniqator Q700 immersion homogenizer (Qsonica, Newtown, CT, USA) with a power of *N*_US_ = 250 W for *t*_US_ = 30 min. To prevent boiling, cooling was applied during processing. As a result, the temperature itself did not exceed 40 °C during mixing. The achieved values of the *F*_US_ energy density during high-intensity ultrasound treatment were estimated using the formula [81]:

(1) $$F_{\mathrm{US}}=\frac{N_{\mathrm{US}}t_{\mathrm{US}}}{V}.$$

According to Formula (1), the *F*_US_ values were 11,250 J/cm^3^. This made it possible to prepare a stable, dispersed SWCNT medium for which sediment formation is not observed.

At the final stage, all the initial components were mixed. Also, the addition of water-soluble eosin-Y (Agat-Med, Moscow, Russia) was performed, while only the amount of gelatin varied. As a result, six samples were obtained. They included a BSA of 20 wt. %, eosin-Y 0.05 wt. %, SWCNTs 0.02 wt. %, and the amount of gelatin for each sample was as follows: 1—3 wt. %, 2—2 wt. %, 3—1.5 wt. %, 4—1 wt. %, 5—0.5 wt. %, 6—0 wt. %. At the specified concentration of nanotubes, uniform homogenization is ensured, which was disrupted for larger concentrations.

**Figure 7:**
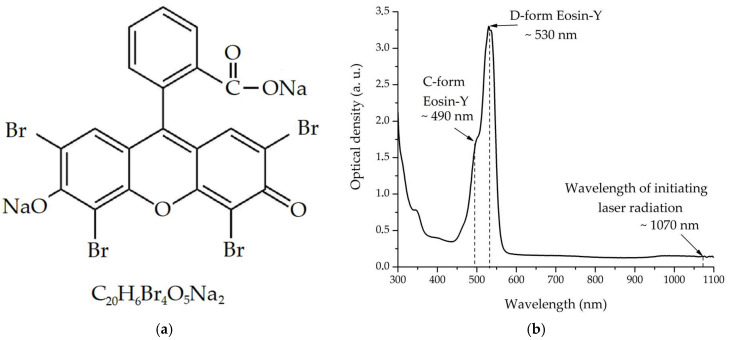
(**a**) The structure of the eosin-Y molecule, and (**b**) the one-photon absorption spectrum of sample 1 prepared with it.

A GENESYS 50 UV-Vis-Nir spectrophotometer (Thermo Fisher Scientific, Waltham, MA, USA) was used to estimate the optical density of the obtained dispersed hydrogel media. The spectrum was recorded at wavelengths between 300 and 1100 nm, with an increment of 2 nm. During the research, a quartz cuvette with an optical path length of d = 1 mm was used. Figure 7 shows data for sample 1 containing eosin-Y (Figure 7a) as a photoinitiator. In the two-photon absorption window (520–540 nm) for the 1070 nm laser line, the values of the linear absorption coefficient α reaches 75 cm^−1^, which indicates that this spectral range is of a significant sensitivity. At the excitation wavelength, the α values are less than 2 cm^−1^, which indicates insignificant one-photon absorption of such radiation. The spectrum is dominated by the absorption bands of eosin-Y (Figure 7b) associated with dianions of the D-form of eosin (λ_max_~530 nm) and its cations of the C-form (λ_max_~490 nm).

### 5.2. Determination of the Two-Photon Absorption Cross Section

**Figure 8:**
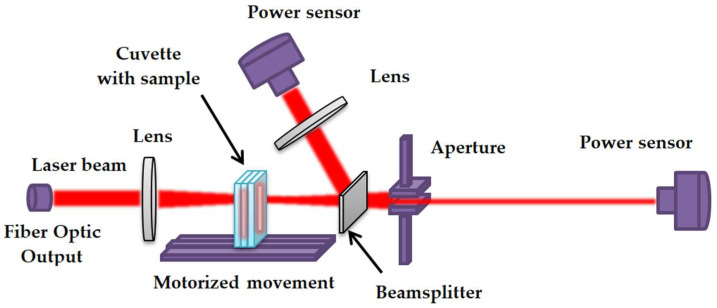
The scheme of the experiment for determining the value of the two-photon absorption cross section.

In order to estimate the two-photon absorption cross section, an optical scheme was constructed that allows measurements using the Z-scan method. Figure 8 shows this configuration. The HTTP MARK MOPA laser system (Bulat Design Bureau, Moscow, Zelenograd, Russia) based on an ytterbium fiber laser was used to generate pulses. The pulses emitted by the laser passed through a lens to focus the radiation and achieve intensity sufficient for the manifestation of nonlinear effects. The sample was then placed in a holder fixed on a motorized stage N31.100E (CoreMorrow Ltd., Harbin, China) with a controller E71.D4E-H (CoreMorrow Ltd., Harbin, China) to move it along the direction of the beam up to the location of the second lens. Subsequently, an aperture is positioned behind the aforementioned component, after which a G5F-GT-10 power sensor (ShenZhen CaiHuang Thermoelectricity Technology Co. Ltd., Shenzhen, China) is installed.

Using the prepared optical scheme, the measurements using the Z-scan method with an open and closed aperture are performed. Using it, estimations of the two-photon absorption cross section were carried out for all six prepared samples. In order to determine its value, an analytical solution of the radiation transfer equation (RTE) [82] was used, which was obtained in approximations of the rectangular pulse shape and taking into account the presence of an energy threshold.

The two-photon absorption cross section, σ_tpa_, describes the efficiency of the transition of the eosin molecule to the excited state and is related with the value of the nonlinear absorption coefficient, which is typically determined from the Z-scan experiment [83]. Based on these considerations, the following formula is derived:

(2) $$T_{norm}=\exp\left(\frac{d\sigma_{\mathrm{tpa}}N_{A}C\cdot 10^{-3}}{\tau E_{\mathrm{ph}}\sqrt{\pi }}\left(F_{\mathrm{th}}-\frac{2U_{0}z_{0}}{\pi w_{0}^{2}\left(z_{0}^{2}+z^{2}\right)}\right)\right).$$

where *d* is the sample thickness; *N*_A_ is the Avogadro number; *C* is the mole concentration divided per liter; τ is the pulse duration; *E*_ph_ is the energy of the absorbed photon; *F*_th_ is the threshold exposure of laser radiation; *U*_0_ is the total energy of the incident pulse; *z* is the sample displacement relative to the lens focus; *z*_0_ is the Rayleigh length; and *w*_0_ is the radius of the beam in the waist.

The nonlinear refractive index was determined based on the results of two measurements using the Z-scan method. Its value is obtained from the data of the normalized transmission ratio for the case with a closed aperture for measurements of this value in the case with an open aperture. In this way, the contribution from nonlinear absorption is taken into account. Finally, the following formula [84] was used:

(3) $$T_{нoрм}=1-\frac{8\pi n_{2}I_{0}\left(1-\exp(-\alpha d)\right)x}{\lambda \alpha \left(x^{2}+9\right)\left(x^{2}+1\right)}.$$

where α is the linear absorption coefficient; *n*_2_ is the nonlinear refractive index; *I*_0_ is the peak intensity in the waist; λ is the wavelength of laser radiation; *x = z/z*_0_ is the normalized coordinate.

### 5.3. Assessing the Biocompatibility

For in vitro studies of the obtained biocomposites, the Neuro 2A cell line, which was acquired at the National Research Center for Epidemiology and Microbiology of the Ministry of Health of the Russian Federation, was used. The studies were conducted on polymerized samples on cover glasses from dispersion 1 with the number of layers of 5, 4, 3, and 2, from dispersions 2 and 3 with the number of layers of 5. Before the experiments, the samples were sterilized with ultraviolet light and washed with cell medium. Cells were cultured in DMEM and 90% culture medium supplemented with 10% calf fetal serum in a 12-well plate. Biocomposites of the formed hydrogels and control samples, which were clean cover glasses, were placed on the bottom of the wells of the plate and filled with cell suspension. The cell concentration was counted using an automatic counter Scepter Millipore (Merck KGaA, Darmstadt, Germany). The cell seeding dose was 2.7·105 cells/ml. After seeding, the cells were incubated with CO_2_ in a thermostat at 37 °C and 5% CO_2_ for 72 h. At the end of the cultivation, the cells were quantified (MTT test), and cell morphology was assessed using fluorescence microscopy. The MTT test was carried out according to the standard method [85]. Optical density readings were performed using an Immunochem-2100 microplate photocalorimeter (High Technology Inc., North Attleboro, MA, USA), at a wavelength of 492 nm. Cells were stained for fluorescence visualization using a Hoechst 33,342 dye (Life Technologies, New York, NY, USA) at 10 mg/mL. Cells with dye were incubated for 15 min at 37 °C in a CO_2_ thermostat. Cells on the samples were viewed on an Olympus FV3000 microscope (Olympus Corporation, Tokyo, Japan) using the FV31S-SW viewer software v2.3 (Olympus Corporation, Tokyo, Japan).

## Figures and Tables

**Table 1:** Comparison of the characteristics of electrically conductive hydrogels used for neural tissue engineering.

Composition of Hydrogel	Conductivity, mS/cm	MTT Assay	Reference
PANi/polyethyleneglycol diacrylate (PEGDA)	0.001	0.4–0.8	[22]
PCL/chitosan	0.21	0.1–0.5	[23]
nano graphene oxide/chitosan	1	0.9–1.1	[24]
PPy/chitosan	1.3	0.4–0.6	[25]
PPy nanoparticles/collagen	2.2	0.4–1.2	[26]
PEDOT/chitosan	4.68	0.7–0.8	[27]
CNT/poly(L-lactic acid)	6	0.4–1	[28]
PANi/graphene (PAG)/chitosan/gelatin matrix	0.1–10	0.4–0.85	[29]
gelatin-graft-PANi/periodateoxidized alginate/polyethylenimine	3.12–13.4	1–1.1	[30]

**Table 2:** Properties of hydrogels.

Hydrogels	Percentage by Weight of Gelatin ω_g_, wt. %	Linear Absorption Coefficient α, cm^−1^	Two-Photon Absorption Cross Section σ_tpa_, GM	Threshold Exposure of Laser Radiation Fth, J/cm^2^	Linear Refractive Index n_0_, a.u.	Nonlinear Refractive Index n_2_, 10^−12^ cm^2^/W	Dynamic Viscosity Coefficient η, mPa∙s
1	3 ± 0.2	1.8 ± 0.1	580 ± 70	0.03 ± 0.01	1.372 ± 0.001	42 ± 2	4.8 ± 0.1
2	2 ± 0.2	1.8 ± 0.1	470 ± 70	0.04 ± 0.01	1.371 ± 0.001	40 ± 2	4.6 ± 0.1
3	1.5 ± 0.1	1.9 ± 0.1	440 ± 50	0.04 ± 0.01	1.369 ± 0.001	38 ± 2	3.6 ± 0.1
4	1 ± 0.1	1.8 ± 0.1	320 ± 30	0.05 ± 0.01	1.367 ± 0.001	32 ± 2	2.8 ± 0.1
5	0.5 ± 0.1	1.9 ± 0.1	260 ± 30	0.05 ± 0.01	1.365 ± 0.001	31 ± 2	2.3 ± 0.1
6	0	1.6 ± 0.1	160 ± 30	0.06 ± 0.01	1.361 ± 0.001	30 ± 2	1.9 ± 0.1

**Table 3:** Electrical conductivity of samples.

Biocomposites	Number of Layers	Specific Resistance, Om·cm	Specific Conductance, mS·cm^−1^
1	5	53 ± 5	19 ± 2
	4	59 ± 5	17 ± 2
	3	63 ± 7	16 ± 2
	2	76 ± 7	13 ± 2
2	5	55 ± 5	18 ± 2
3	5	650 ± 50	2 ± 1

## Data Availability

Data underlying the results presented in this paper are not publicly available at this time but may be obtained from the corresponding authors upon reasonable request.
